# Engineered Mitochondrial Ferritin as a Magnetic Resonance Imaging Reporter in Mouse Olfactory Epithelium

**DOI:** 10.1371/journal.pone.0072720

**Published:** 2013-08-30

**Authors:** Bistra Iordanova, T. Kevin Hitchens, Clinton S. Robison, Eric T. Ahrens

**Affiliations:** 1 Department of Biological Sciences, Carnegie Mellon University, Pittsburgh, Pennsylvania, United States of America; 2 Pittsburgh NMR Center for Biomedical Research, Carnegie Mellon University, Pittsburgh, Pennsylvania, United States of America; 3 Department of Radiology, University of California San Diego, San Diego, California, United States of America; Wayne State University, United States of America

## Abstract

We report the design of a MRI reporter gene with applications to non-invasive molecular imaging. We modified mitochondrial ferritin to localize to the cell cytoplasm. We confirmed the efficient cellular processing of this engineered protein and demonstrated high iron loading in mammalian cells. The reporter’s intracellular localization appears as distinct clusters that deliver robust MRI contrast. We used this new reporter to image *in vivo* and *ex vivo* the gene expression in native olfactory sensory neurons in the mouse epithelium. This robust MRI reporter can facilitate the study of the molecular mechanisms of olfaction and to monitor intranasal gene therapy delivery, as well as a wide range of cell tracking and gene expression studies in living subjects.

## Introduction

Complex and opaque systems such as the brain present unique challenges for visualizing molecular events. The primary sensory neurons are the first step on the pathway of transforming the physical world into cortical representations that ultimately drive behavior. For example, the sense of smell is initiated by binding of an odorant to receptors expressed on olfactory sensory neurons (OSN) in the nasal olfactory epithelium (OE) [1]. The OSN carry the sensory information from the nose to the brain, and significant effort has been invested in the last two decades to better understand the molecular biology and the genetics of these cells [1], [2]. A major hurdle for mapping the molecular routes of olfaction is the absence of reliable exogenous model systems [3]. The majority of studies rely on laborious *post mortem* methods to probe the OSN morphology, and the dynamics of the odorant receptors (OR) has to be reconstructed from snapshots of individual time points [1], [2]. The use of reporter genes has been instrumental for the visualization of olfactory maps [4], as well as for the functional expression of OR [5]. Progress in the field has been slow, however, since the bone enclosure and the complex topology of the OE greatly impede *in vivo* experiments using optical reporters.

Magnetic resonance imaging (MRI) is not limited by opacity of deep tissues; it is inherently three dimensional, non-invasive, and well suited for longitudinal studies. Blood oxygen level-dependent (BOLD) MRI is widely used for functional mapping of odors [6] and manganese enhanced MRI was recently used to detect individual glomeruli [7]. The direct study of molecular events in the brain; however, requires the use of reporters that can be expressed in molecularly-defined subpopulations of cells or in tandem with genes of interest. Due to its relatively low sensitivity [8], MRI detection of gene expression is still in its infancy.

The iron storage protein, ferritin, has gained attention as an MRI reporter because of its ability to induce image contrast without the need for small molecule substrates or nanoparticles. Upon cellular expression, ferritin sequesters iron *in situ* to form a superparamagnetic core that creates local magnetic field perturbations and image hypointensity within T_2_*-weighted MRI [9]. Ferritin was successfully used as a ‘probeless’ MRI reporter gene in both vector mediated gene delivery and transgenic animal models [10]–[14].

Optimization of ferritin contrast efficacy is feasible using molecular biotechnology tools. We recently engineered a ferritin chimera with improved sensitivity [15] and used it to visualize native neuroblast migration towards mouse olfactory bulb [16]. Mitochondrial ferritin (FTMT) was recently identified by database searches as an intronless gene with 80% similarity to the cytosolic heavy chain ferritin (FTH) [17]. The iron storage capacity of FTMT is similar to the ubiquitous FTH, however FTMT has greater avidity for iron [18]. When FTMT is expressed as a transgene in a cell line, it loads more iron than FTH [19]. In addition, FTMT is more stable, resisting guanidine and heat denaturation compared to FTH [20], and thus potentially a more robust reporter molecule.

Here, we report the design of a modified FTMT with localization into the cell cytoplasm (cytoFTMT) that displays high iron loading efficiency. We use this new MRI reporter to image *in vivo* gene expression in native mouse OSN. This technology platform can be used to report on molecular events deep into opaque brain tissues and may be useful in broad spectrum of neurobiology applications.

## Materials and Methods

### cDNAs and Viral Vectors

Full length and cytoplasmic (cyto) mitochondrial ferritin (FTMT) cDNAs were produced using standard techniques and oligonucleotide-mediated PCR mutagenesis. FTMT was cloned from human genomic DNA as previously described [17]. The major portion of the mitochondrial localization leader (194 nucleic acids) was truncated to obtain the cytoFTMT with resulting cytoplasmic localization [19].

To express transgenes, we constructed replication deficient, type 5 adenovirus (AdV5, deleted for E1/E3) expression vectors, based on the BD Adeno-XTM Expression System 1 (BD Biosciences Clontech, Carlsbad, CA, USA). Briefly, the cDNAs were inserted into appropriate restriction sites in pShuttle-2 and then subcloned into pAd-X plasmid to generate the recombinant AdV.

We recovered, isolated and propagated viral stocks using human embryonic kidney cells (HEK-293, ATCC, Manassas, VA, USA, #CRL-1573). Working stocks of virus were produced using double CsCl density gradient purification. The final purified vector preparations were dialyzed using TNMG buffer (10 mM Tris, 150 mM NaCl, 1 mM MgCl2, 5% glycerol). The recombinant AdV5 viruses were titered on HEK293 cells using immunodetection for single infectious units. The titers, in units of pfu/mL, were as follows: cytoFTMT AdV 2.6×10^11^, FTMT 1.9×10^11^, lacZ AdV 3.7×10^10^, L*H AdV 2×10^10^, and FTH AdV 1.4×10^10^. The eGFP AdV used had a titer of 1×10^11^ pfu/mL and was purchased from the University of Pittsburgh Vector Core Facility.

### Cell Line and Western Blot

Human osteosarcoma cells U2OS (ATCC, #HTB-96) were grown and maintained in complete medium (90% DMEM, 10% FBS) and transduced with cytoFTMT, FTMT and lacZ AdV using a multiplicity of infection (MOI) of 30. Cells expressing the different ferritins and control reporter (lacZ) were harvested at 48 h post-transduction and lysed in a detergent solution containing protease inhibitor cocktail and EDTA (Pierce, Rockford, IL, USA). Total protein content was measured with a bicinchoninic acid (BCA) assay kit (Pierce, Rockford, IL). Equivalent amounts of clarified samples were resolved on 4–20% polyacrylamide gradient gels (Pierce). Primary antibody used for the western blot analysis was mouse monoclonal to mitochondrial ferritin (ab55027, Abcam, Boston MA). We used chemiluminescence to expose the immunoreactive bands on film (1651454, Eastman Kodak, Rochester, NY). The gels were stripped and reprobed with mouse monoclonal for β-actin (sc-47778, Santa Cruz). Secondary antibodies were goat anti-rabbit horseradish peroxidase (HRP) conjugate (1858415, Pierce, Rockford, IL) and goat anti-mouse HRP conjugate (1858413, Pierce).

### Inductively Coupled Plasma Emission Spectroscopy (ICP-ES)

We transduced U2OS cells with AdV coding for the different ferritins and lacZ control using MOI of 30. At 24 hours post-transduction, the culture media was supplemented with 1 mg/ml holotransferrin (T0665, Sigma-Aldrich, St. Louis, MO) and 1 mM ferric citrate (F3388, Sigma-Aldrich). At 48 hours post-transduction all culture media was aspirated and cells were washed, harvested and counted.

For cell digestion, the cells were spun down in Oak Ridge centrifuge tubes (3119-0010, Nalgene), resuspended in 70% nitric acid and heated in an autoclave with vacuum for moisture removal for 1 hour at 90°C. The dried and digested material was then resuspended in 5 ml of 1% nitric acid, and the tubes were agitated in an ultrasonic water bath for one hour to bring the cell pellets into solution. We used a Perkin Elmer Optima 3000DV ICP (Perkin Elmer, Waltham, MI) in axial mode for the analysis. The results are obtained as mg/L (ppm) in the solutions and then normalized by cell number. The detection limit for iron on this instrument is 0.003 ppm.

### Cytotoxicity Assay

We used the Vybrant cytotoxicity assay (V-23111, Molecular Probes, Eugene, OR) according to the manufacturer’s instructions. We measured the amount of released enzyme glucose 6-phosphate dehydrogenase (G6PD) of U2OS cells 48 hours after the AdV transduction with the different ferritin transgenes and lacZ control. The fluorescence was measured with a Tecan Safire 2 fluorescence plate reader (Tecan Group Ltd., Durham, NC) using excitation/emission ∼530/590 nm. A background fluorescence value of the cell medium alone was subtracted from each value. The values were normalized to the fluorescence of lysed cells representing the total cellular G6PD. For positive control, we used 50 µM camptothecin (C9911, Sigma-Aldrich).

### Statistical Analysis

The significance of the ICP-ES results and the cytotoxicity assay was calculated using Origin 7.5 software (Northampton, MA). We used one-way analysis of variance (ANOVA) to look at differences of the group means, followed by Tukey’s method for pair-wise differences with a confidence interval of 0.95.

### Immunocytochemistry and Subcellular Localization Study

U2OS cells were grown on 35 mm glass bottom cell culture dishes (WillCo 64-0758, Warner Instruments, Hamden, CT) and transduced with reporter transgenes. At 48 h post-transduction, cells were fixed using 4% paraformaldehyde (PFA). The cells were washed with phosphate buffer saline (PBS) and 0.2% Tween 20 (Bio-Rad, Hercules, CA) and then probed using antigen-specific antibodies followed by the secondary reagents as described below. We used mouse monoclonal to mitochondrial ferritin (ab55027, Abcam), MitoTarcker Orange (M7510, Invitrogen, Carlsbad, CA), mouse monoclonal to LAMP2, lysosome marker (ab25631, Abcam), and rabbit polyclonal to Golgi protein marker GOLH4 (ab28049, Abcam). Cell nuclei were counterstained with Hoechst 3342 (Calbiochem, La Jolla, CA).

### *In Vivo* Studies

All animal experiments were approved by the Carnegie Mellon Institutional Animal Care and Use Committee (IACUC). Adult female (N = 10) C57BL mice (Harlan, Indianapolis, IN), 5–7 weeks old, were anesthetized with an intraperitoneal cocktail of ketamine/xylazine. The mice were placed supine and a cannula was inserted into the nasal cavity approximately 7 mm deep. A viral vector solution (10 µl) was slowly administered intranasally over 5–10 minutes. One nasal passage received cytoFTMT AdV and the opposite nasal passage received the eGFP AdV control. The mice remained supine for at least 15 minutes post-administration to aid vector absorption to the olfactory epithelium. Mice were intubated prior to this procedure to maintain a clear airway. Approximately 48 h after the viral administration, iron supplement solution of 10 mM ferric citrate (Sigma, #F3388) and 2 mg/ml holotransferrin (#T0665, Sigma) was introduced to both nasal passages by the same intranasal instillation procedure. At 48 hours later, the mice were imaged. After imaging, animals were perfused transcardially with PBS and then with 4% PFA, and the brains were either embedded in paraffin or flash frozen in optimal cutting temperature (OCT) compound (EMS, Hatfield, PA) and stored at −80°C.

### MRI

*In vivo* MRI was performed at 7 T using a Bruker Biospec imaging system (Bruker, Billerica, MA). The mice were anesthetized, intubated, placed on a mechanical ventilator, and maintained on 0.75% isoflurane in 70% O_2_ and 30% N_2_O inhalation gas during the imaging sessions. For *in vivo* imaging we used a quadrature receive mouse brain surface coil (Rapid Biomedical, Columbus, OH) and for excitation we used a 72 mm birdcage coil (Bruker). T_2_*-weighted images were acquired using a 3D gradient-echo (GRE) sequence with TE/TR = 7/100 ms, 20° flip angle, 4 averages, 128×128×128 matrix, field of view = 1.5×1.5×1.5 cm and 117 µm isotropic resolution. The total *in vivo* scan time was 1 hour 49 min. For the *ex vivo* imaging, we used an 11.7 T Bruker microimaging system with a volume coil and a 3D GRE sequence with TE/TR = 8/100 ms, 20° flip angle, 4 averages, 256×256×256 matrix, field of view = 1.28×1.28×1.28 cm and 50 µm isotropic resolution. The total *ex vivo* scan time was 7 h 16 min.

### Immunohistochemistry and Histology

Frozen brain tissue was cryosectioned in 20 µm thick slices, and paraffin embedded tissue was sectioned into 10 µm thick slices. All tissue sections were mounted on glass slides. The slides were incubated for one hour at room temperature with primary antibodies in a humidified chamber, washed and incubated for 45 min with secondary antibodies. We used rabbit polyclonal antibody to Olfactory Marker Protein (OMP) (ab62609, Abcam) and mouse monoclonal antibody to mitochondrial ferritin (ab55027, Abcam), with secondary Alexa Fluor 594 F(ab’)2 fragment of goat anti-rabbit (A1172, Invitrogen), goat anti-rabbit Alexa Fluor Alexa 635 (A31576, Invitrogen) and Alexa Fluor 568 F(ab’)2 fragment of goat anti-mouse (A11019, Invitrogen). Nuclei were counterstained with Hoechst 3342 (Calbiochem). Control sections were incubated with the secondary antibodies without the presence of the primary antibody. For the Perls’ iron stain the sections were immersed in water with 2% potassium ferrocyanide and 2% concentrated hydrochloric acid for 30 min. Cell nuclei were counterstained with 0.5% nuclear fast red (H-3403, Vector Labs, Burlingame, CA). Paraffin embedding, sectioning and Hematoxylin & Eosin (H&E) staining was performed by AML Laboratories (Baltimore, MD).

### Confocal Microscopy

Glass bottom cell culture dishes and glass cover slips were mounted after the final wash and were imaged using a Carl Zeiss LSM 510 Meta UV DuoScan inverted confocal laser-scanning microscope. RGB channels were collected sequentially with a matrix resolution of 1024×1024 and 4 averages.

## Results

### Reporter Gene Constructs and Subcellular Localization

**Figure 1:**
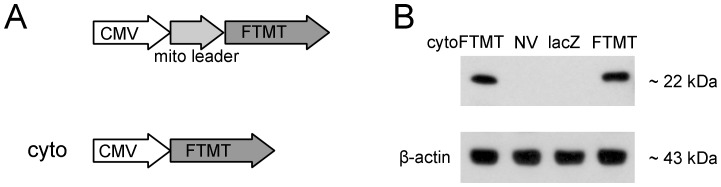
MRI reporter gene construct and its expression. (**A**) Top, mitochondrial ferritin (FTMT) gene with intact mitochondrial targeting leader was expressed under cytomegalovirus (CMV) promoter. Bottom, the mitochondrial targeting leader was truncated from an isogenic FTMT construct to achieve cytoplasmic localization (cytoFTMT). (**B**) Western blotting showing FTMT expression in U2OS cells 48 hours post-transduction with adenoviral vectors expressing the two constructs.

We cloned the human mitochondrial ferritin (FTMT) gene from human blood. To obtain cytoplasmic localization for the new reporter molecule (cytoFTMT), we truncated 64 residues from the polypeptide leader that contains the mitochondrial targeting signal [17]. Figure 1A shows a schematic of the two constructs, the native FTMT and the truncated cytoFTMT. We sequenced both constructs and confirmed their nucleotide fidelity by aligning them to the published human FTMT gene sequence [17] (Figure S1 in File S1). The FTMT protein is initially synthesized as a 30 kDa peptide that includes the targeting precursor, and upon delivery to the mitochondria the protein is proteolytically processed into a 22 kDa peptide [17]. In order to verify the size of the polypeptide chains resulting from the transgene expression, we transduced a mammalian cell line (U2OS) with adenovirus (AdV) coding for FTMT and cytoFTMT under the cytomegalovirus (CMV) promoter. As predicted, the resulting bands for both proteins were approximately 22 kDa (Figure 1B).

**Figure 2:**
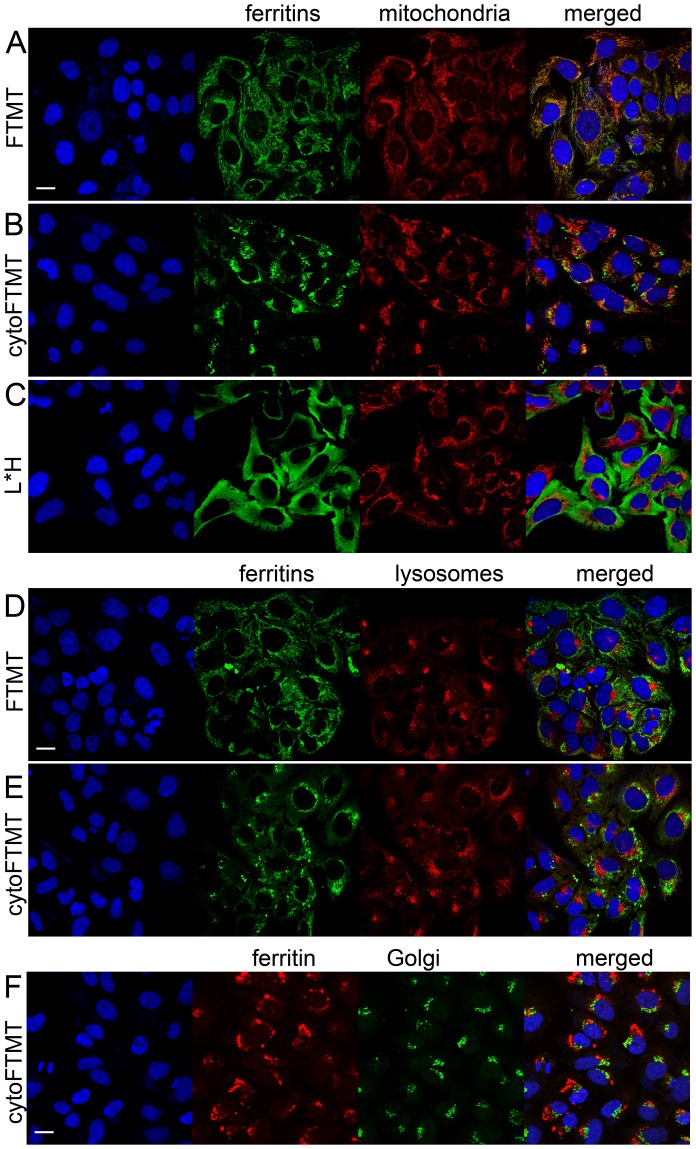
Confocal images of U2OS cells showing colocalization of the transgene reporters with cellular organelles in the protein processing pathway. (**A**) FTMT (green) with intact mitochondrial targeting localizes to the cellular mitochondria (red). (**B**) cytoFTMT (green) forms aggregates and has low-level colocalization with mitochondria (red). (**C**) By comparison, the L*H ferritin chimera (green) displays diffuse cytoplasmic distribution. (**D**) FTMT (green) does not colocalize with the lysosomes (red). (**E**) cytoFTMT (green) does not colocalizes with lysosomes (red). (**F**) cytoFTMT (red) is not retained in Golgi (green). Nuclei are stained with Hoechst blue. Scale bar = 20 µm.

Next, we investigated the reporter protein localization in cells. As expected from the intact mitochondrial targeting, the distribution of FTMT had mitochondrial phenotype (Figure 2A). Unexpectedly, the cytoFTMT showed pronounced aggregation, in addition to the anticipated diffuse cytoplasmic staining (Figure 2B). To confirm that the aggregation of cytoFTMT protein is not simply an artifact from the viral expression or idiosyncrasy of the specific cell line, we used the same expression system to visualize the distribution of FTH and previously described L*H ferritin chimera [15]. As expected, the FTH had diffuse cytoplasmic and nuclear localization [21] (Figure S2A in File S1), and in accordance with previous results [15], the L*H ferritin chimera had primarily a cytoplasmic distribution (Figure S2B in File S1). These controls, as well as the expression of lacZ control and no virus control (Figs. S2C and S2D in File S1) supported the observation that punctate protein aggregation was specific to the cytoFTMT.

To further assess the subcellular distribution of the reporter proteins, we looked at their colocalization with several cytoplasmic organelles on the protein processing pathway. First, we confirmed that FTMT colocalizes with cellular mitochondria (Figure 2A). Due to the truncated targeting signal, there was very weak colocalization between the cytoFTMT and the cellular mitochondria (Figure 2B). It was apparent, however, that cytoFTMT aggregates in the vicinity of the mitochondrial network and its cytosolic presence differed considerably from the diffuse distribution of the L*H ferritin chimera (Figure 2C). Cells transduced with lacZ control and stained for mitochondria can be seen in Figure S3A in File S1.

We next investigated whether the aggregation of cytoFTMT was a consequence of a protein misfolding and a subsequent degradation. The common degradation pathway for ferritins is via lysosomal proteolysis [22], and thus we looked for the presence of the reporter molecule in the cellular lysosomes. We found no significant incidence of either FTMT or cytoFTMT in the lysosomes (Figure 2D–E). In addition, we established that cytoFTMT is not retained in Golgi instead of being exported into the cytoplasm (Figure 2F). The FTH, L*H and lacZ controls for these colocalization experiments are shown on Figure S3B–F in File S1.

### Reporter Molecules Load Iron Efficiently and are Non-toxic

**Figure 3:**
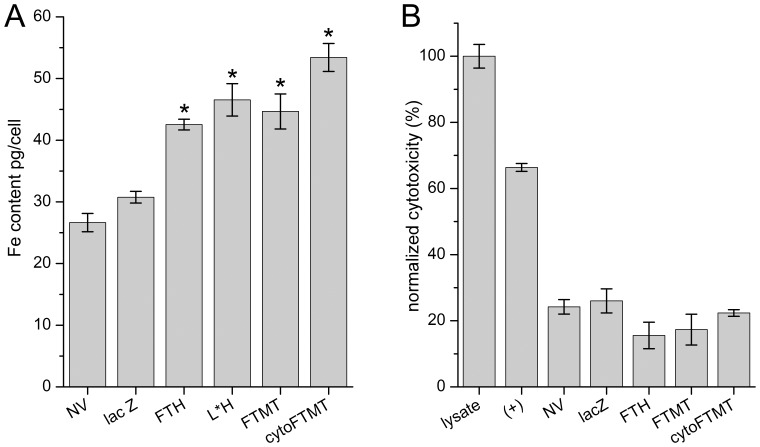
Ferritin reporters load iron efficiently and are non-toxic to cells. (**A**) Inductively coupled plasma emission spectroscopy (ICP-ES) of U2OS cells expressing ferritin transgenes and grown in iron-rich media. Cells expressing all ferritins have significantly more iron than lacZ control and no vector (NV) control. The cytoFTMT transgene loads more iron than FTH (ANOVA = 0.00008, followed by Tukey test for means comparison). (**B**) Cytotoxicity assay showing glucose-6-phosphate dehydrogenase (G6PD) enzyme activity in U2OS cells. Lysed cells represent 100% toxicity. Positive control (+) is cells incubated with the cytotoxic alkaloid camptothecin. Cells expressing all transgenes (ferritins and lacZ control) do not differ significantly from each other.

We investigated whether the transgene expression of the reporter proteins results in a functional iron storage molecule. We used inductively coupled plasma emission spectroscopy (ICP-ES) to measure the elemental iron in U2OS cells expressing the different ferritins and lacZ control. In order to provide a sufficient source of iron, the cells were grown in the presence of an iron supplement. All ferritins loaded significantly more iron than lacZ control with the highest levels in cells expressing cytoFTMT (Figure 3A). Interestingly, the cytoFTMT expressing cells contained more iron than cells expressing FTH (ANOVA P = 0.00008, followed by Tukey test for comparison of the means).

We confirmed that reporter gene expression does not overtly affect cell viability. We measured the activity of glucose-6-phosphate dehydrogenase (G6PD), an assay reflecting cellular health and energy metabolism. We found no difference in G6PD activity between non-transduced cells, cells expressing lacZ and all ferritins. In contrast, both lysed cells and cells treated with the cytotoxic alkaloid camptothecin showed pronounced cytotoxicity (Figure 3B).

### Mouse OE Transduced with cytoFTMT Displays Pronounced MRI Contrast

**Figure 4:**
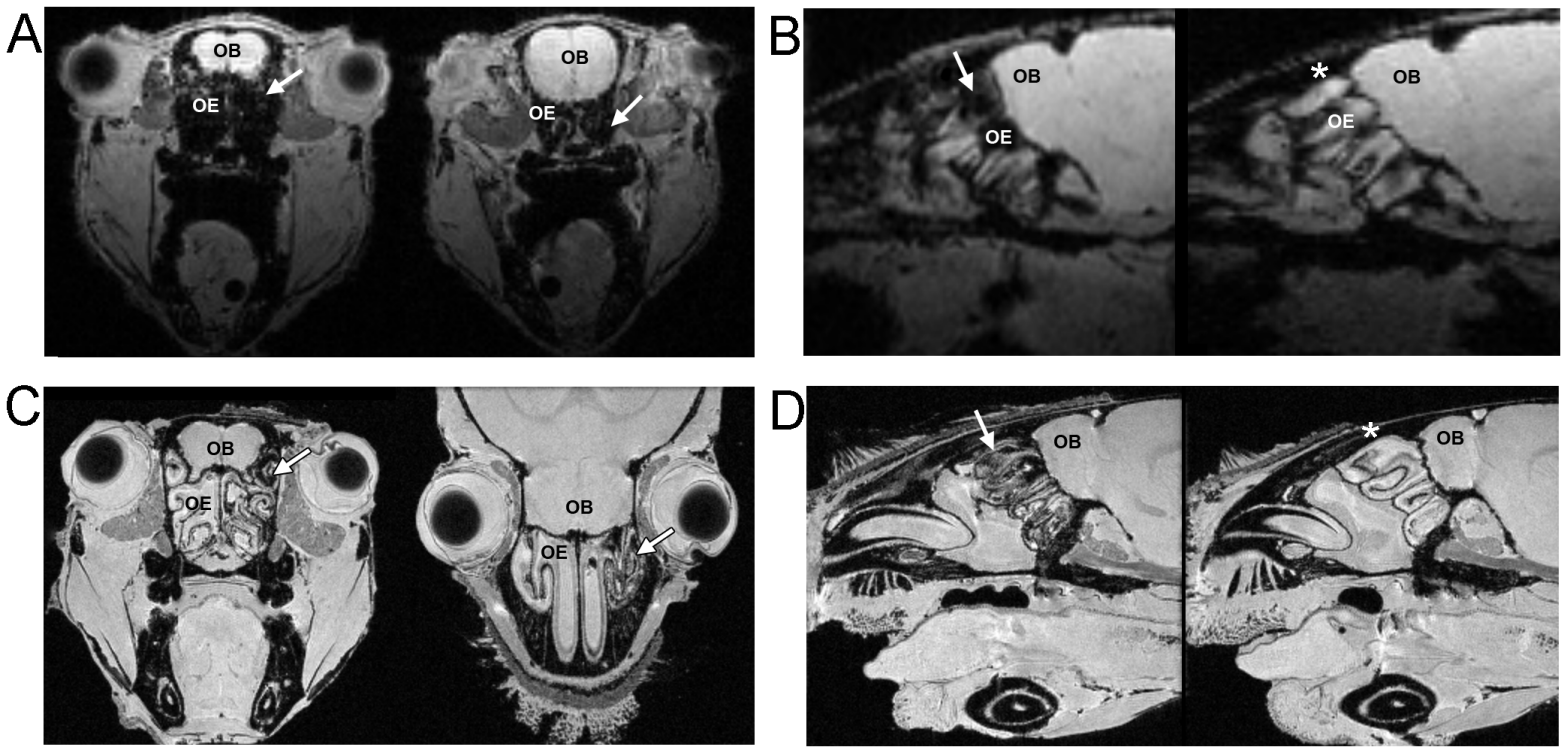
MRI showing expression of cytoFTMT reporter in mouse olfactory epithelium (OE). (**A**) *In vivo* MRI with 117 µm isotropic resolution showing the olfactory bulb (OB) and hypointensity in the OE (arrow) at the site of MRI reporter expression. (**B**) *In vivo* sagittal slices from a different mouse showing the extent of hypointensity in the reporter cytoFTMT side (arrow) and no change in contrast in the lacZ control side (asterisk). (**C**) Axial and coronal *ex vivo* MRI with 50 µm isotropic resolution. Arrows point to the cytoFTMT reporter side. Contralateral side is inoculated with lacZ control and shows no change in contrast. (**D**) *Ex vivo* sagittal slices from a different mouse showing the extent of hypointensity in the reporter cytoFTMT side (arrow) and no change in contrast in the eGFP control side (asterisk).

We next pursued one of the main goals of our study, that is, to be able to visualize gene expression in sensory neurons in live animals. In the right nostril of a mouse, we introduced an AdV coding for cytoFTMT; the contralateral nostril received AdV eGFP. At 48 hours, iron supplementation solution was administered to both nasal passages. Four days after the viral transduction we imaged the mouse olfactory epithelium *in vivo* and observed pronounced MRI contrast on the side of the ferritin reporter expression (Figure 4A,B, arrows). The contralateral eGFP control side showed no change in MRI contrast (Figure 4B, asterisk). The same contrast features were observed when imaged *ex vivo* at higher spatial resolution. We were able to discern the OSN layer in the OE and higher contrast on the MRI reporter side that originated from this cellular layer (Figure 4C,D, arrows). We were also able to see the cellular layers on the control side; however, the image contrast was considerably less pronounced than the MRI reporter side (Figure 4D, asterisk). Since the mice were kept supine to facilitate the viral inoculum reaching the OE at the posterior portion of the nasal cavity, we observed only modest image contrast at the anterior respiratory portions (Figure S4A,B, arrows, in File S1). There was no difference in image contrast at the vomeronasal organ between the cytoFTMT and eGFP expressing sides since anatomically this organ uses a separate nasal passageway from the main OE [1] (Figure S4A,B, arrowheads, in File S1). We saw robust MRI contrast on the cytoFTMT side along a substantial area of the OE (Figure S4C,D, arrows, in File S1).

### MRI Reporter is Expressed in OSN and their Cell Projections Reach the Brain

**Figure 5:**
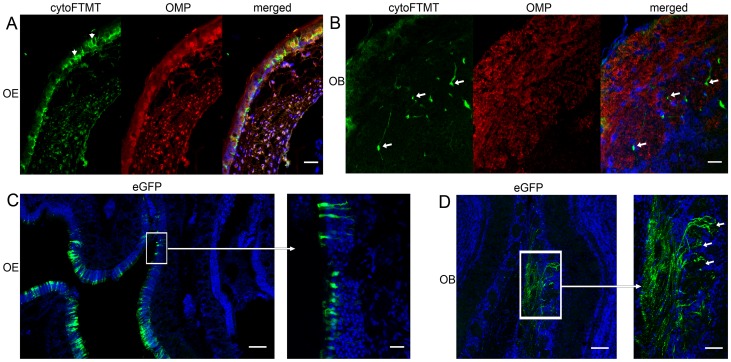
MRI reporter cytoFTMT is expressed in olfactory sensory neurons (OSN) in the mouse olfactory epithelium (OE) and cellular projections reach the olfactory bulb (OB). (**A**) The reporter cytoFTMT (green) displays punctate distribution (arrowheads) in the OE and is expressed in cells positive for olfactory marker protein (OMP) (red). (**B**) Arrows point at projections of the OSN expressing cytoFTMT (green) that reach the glomerular layer in the OB. OMP (red) marker delineates the glomeruli and the OSN axons that come from the OE. Nuclei are stained with Hoechst blue. Scale bar = 20 µm. (**C**) Shows eGFP expression at the contralateral control side of OE. Scale bar = 100 µm. Insert shows detail of the OE, with green cells having OSN morphology. Scale bar = 20 µm. (**D**) The OSN expressing eGFP reach the glomerular layer of the OB. Scale bar = 50 µm. Insert shows detail of the glomeruli and synaptic endings of the OSN (arrows). Scale bar = 20 µm. Nuclei are stained with Hoechst blue.

We established the identity of the cells expressing the reporter and the extent of their cellular processes. Cells at the OE, double stained for cytoFTMT and olfactory marker protein (OMP), displayed both diffused cytoFTMT staining and distinct protein aggregates (Figure 5A, Figure S5A, arrows, in File S1). The aggregates visible on the immunohistochemistry of OSN cells appeared similar to the ones in the U2OS cell line experiments (Figure 2B,E). In addition, we were able to see the end of the projections of cytoFTMT positive cells terminating at the glomeruli of the olfactory bulb (Figure 5B, arrows). There were numerous GFP positive cells with OSN morphology at the contralateral eGFP control side (Figure 5C), and many of their axons were seen at the glomerular layer synapsing with mitral and tufted cells in the bulb (Figure 5D, arrows). We also observed a collection of many green fibers that extended from the olfactory epithelium to the olfactory bulb, forming the olfactory nerve (Figure S5B, asterisk, in File S1).

### The MRI Reporter is not Cytotoxic and it Increases Iron Storage in Tissues

**Figure 6:**
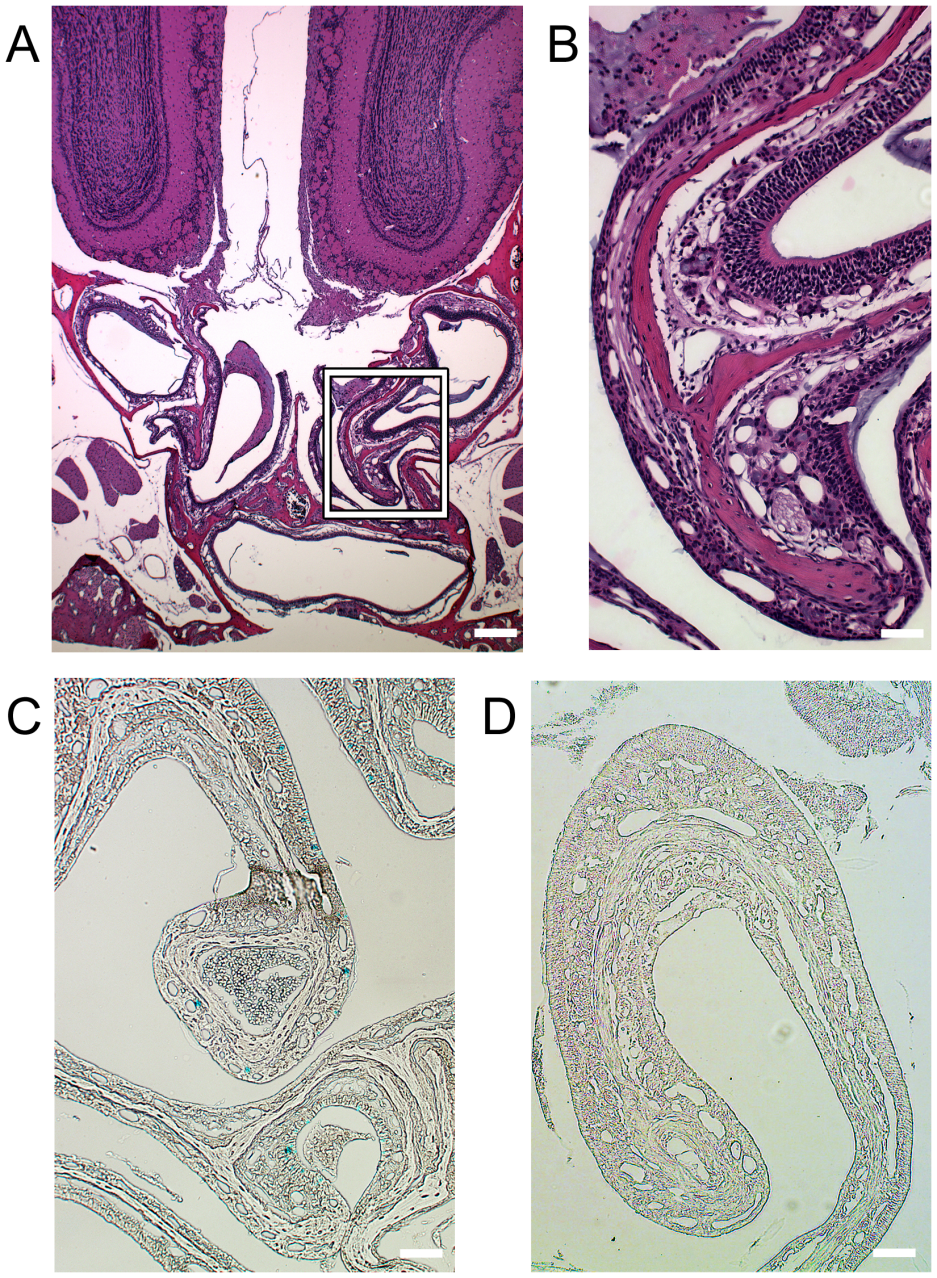
Tissue inoculated with the cytoFTMT reporter shows no visible toxicity and accumulates iron. (**A**) Displays hematoxylin & eosin (H&E) stain of a section including the olfactory epithelium (OE) and the olfactory bulb (OB) of a mouse expressing the MRI reporter on the right side and eGFP on the left. Scale bar = 200 µm (**B**) Insert showing greater detail of OE with normal cellular morphology and no apparent toxicity. Scale bar = 50 µm. (**C**) Perls’ iron stain of section including OB and OE. Asterisk at the control eGFP side of the septum. Scale bar = 200 µm. (**D**) Insert shows magnified view of iron accumulation (blue) in the sensory neuron layer. Scale bar = 50 µm.

There was no apparent tissue cytotoxicity on either the reporter or the control side, and the OE and the bulb had normal morphology of the cellular lamina (Figure 6A,B). The side of the cytoFTMT expression stained positive for iron, indicating that increased iron loading at the site of reporter expression is the source of the MRI contrast (Figure 6C). As expected, the contralateral eGFP control side stained negative for iron (Figure 6D).

## Discussion

We developed a robust approach to visualize noninvasively gene expression in living tissues using MRI. We modified mitochondrial ferritin to localize within the cell cytoplasm, where it can load iron efficiently; this construct functioned as a sensitive MRI gene reporter. We characterized this reporter in mammalian cells and studied the subcellular distribution of its protein aggregates. Using vector mediated expression *in vivo*, we were able to observe gene expression in mouse olfactory epithelium.

The use of ferritin as an MRI reporter is not new, however, use of the recently discovered FTMT gene for MRI has not been previously characterized and yielded surprising results. Compared to FTH, FTMT has distinct biochemical properties such as higher iron avidity [23] and shell stability [20], thus making it a second generation MRI gene reporter molecule with potential sensitivity advantages over prior art. We speculate that the MRI sensitivity of FTMT is increased in part due to the intracellular protein aggregation. However, in our study, putative sensitivity enhancements of FTMT over FTH were only evaluated using *in vitro* data. In divergence with previously reported FTMT expression in cell lines [17], [19], we did not remove the entire localization signal on the FTMT gene, and the remaining portion on the polypeptide chain may be one of the possible reasons for the observed protein aggregation. Under certain conditions, ferritin molecules are known to self-assemble into clusters [24], and the *in vivo* formation of intermolecular aggregates can contribute to paramagnetic enhancement and the intracellular stability of ferritin [25]. Importantly, on its ability to impart contrast, others [26] have demonstrated that induced aggregation of ferritin significantly increases the MRI contrast potency, i.e., its relaxivity per Fe ion.

Using immunocytochemistry and microscopy (Fig. 2), we investigated the major degradation pathways of ferritin and found no evidence of misfolding. However, potentially cellular pathways such as proteasome degradation may be in play. The increased iron loading with cytoFTMT, without overt cytotoxicity, and the resulting MRI contrast point suggest that there is no error in reporter processing.

Sensitivity is one of the major hurdles for molecular MRI. We employed several biotechnology tools to improve detectability. We used high titer purified adenovirus to express the cytoFTMT gene under a strong CMV promoter *in vivo*. AdV does not integrate into the host chromosomes, and its episomal plasmid propagation can deliver very high levels of transgene expression due to a high copy number of plasmid per cell [27]. In addition, AdV is known to preferentially target the olfactory epithelium [28]. Long term studies have demonstrated that AdV mediated transgene expression in OSN can persist for 21–30 days [28], [29]. By this time most of the OSN are reaching the end of their natural life span, and therefore this MRI reporter system is well suited to look at *in vivo* expression of olfactory specific genes such as OR, guidance and signaling molecules.

We have previously shown that an engineered ferritin reporter can be detected *in vivo* at levels on the order of 10^4^ cells [16]. Such detection range is sufficient to tackle a number of molecular questions in olfaction, since many of the OR genes are expressed in populations of at least 50,000 neurons [30]. The molecular regulation of OR gene choice, for example, is still a topic of intense investigation [31], [32]. MRI reporter gene expressed under OR promoter or a regulatory element of interest could facilitate the study of the spatial and temporal landscape of gene expression in the OE. In addition, a transynaptic expression of MRI reporter gene from sensory neurons to distant areas of cell clusters such as the amygdala and the piriform cortex could be potentially imaged *in vivo* and may aid the study of cortical maps of sensory input.

Retrograde transfer of viral vectors encoding for an MRI reporter in tandem with a gene of interest is also an attractive system for the development of gene and small peptide therapy of neurological disorders. The OSN-mediated intranasal route to the brain circumvents the blood-brain barrier and presents a unique and non-invasive path for introduction of therapeutic molecules to affected brain tissues [33].

To our knowledge this is the first application of FTMT gene as an MRI reporter and the first observation of gene expression in native sensory neurons using MRI. This approach could potentially enable the non-invasive visualization of gene expression in broad range of neurobiology applications. Other neurobiology applications include, for example, non-invasive study of cell migration during early development, plasticity and cell repair during cell therapy, and promoter-regulated expression in transgenic models. Moreover, improved ferritin-based MRI reporters can be broadly used in a wide range of preclinical cancer and regenerative medicine applications.

## Supporting Information

File S1 **Contains supporting information for this manuscript.**
(PDF)
