# Supplementary material for: Engineered Mitochondrial Ferritin as a Magnetic Resonance Imaging Reporter in Mouse Olfactory Epithelium
Source: PLoS One. 2013 Aug 30;8(8):e72720. doi: 10.1371/journal.pone.0072720 (PMC3758330; doi:10.1371/journal.pone.0072720)
Supplement: File S1 — Contains supporting information for this manuscript. (PDF) [file pone.0072720.s001.pdf]

SUPPLEMENTARY DATA

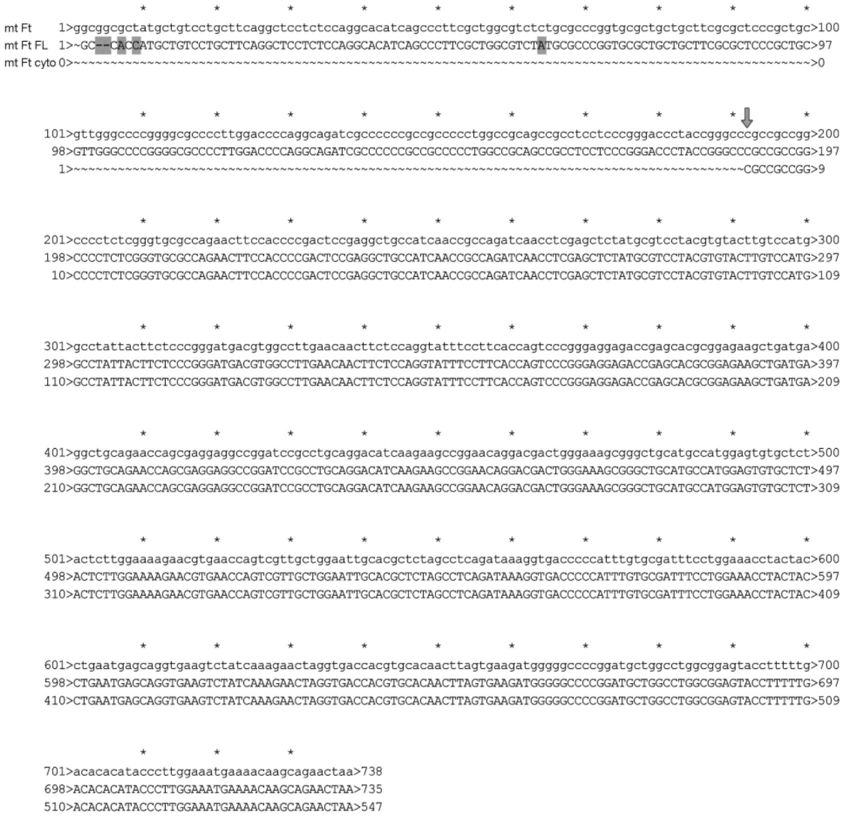

Figure S1. Nucleotide fidelity of the FTMT and cytoFTMT reporter genes. The originally published gene sequence of FTMT (top row) was used as a template to align the full length FTMT (middle row) and the truncated cytoFTMT (bottom row) reporters used in this study. Shadowed areas indicate mismatches due to single nucleotide polymorphisms. The vertical arrow shows the truncation site at the mitochondrial targeting signal of the cytoFTMT.

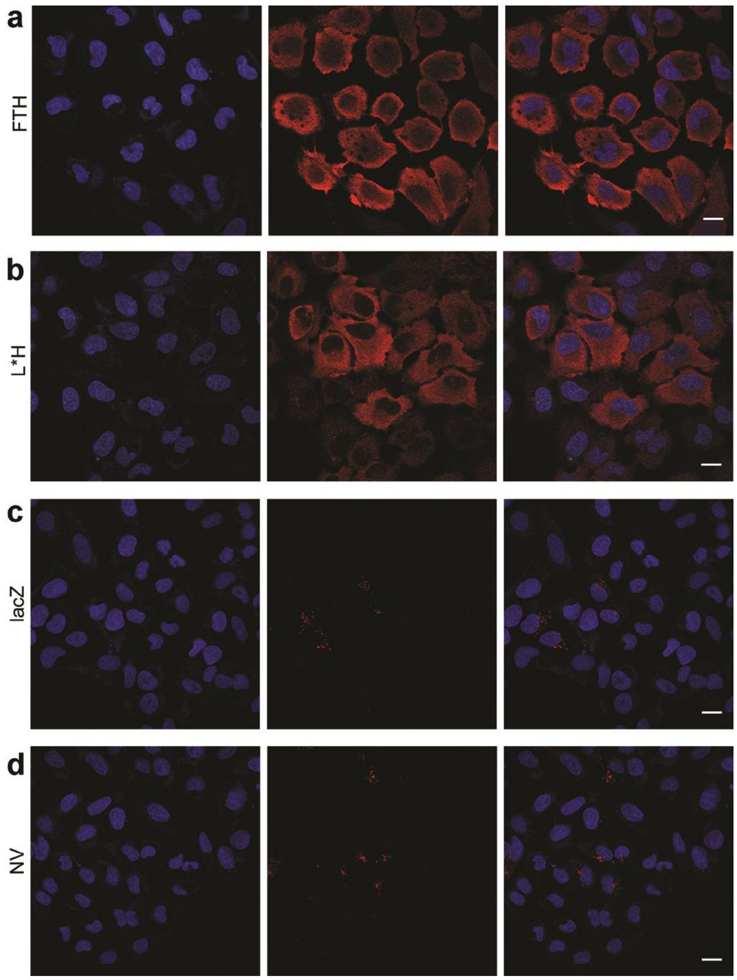

Figure S2. Confocal images of U2OS cells expressing different control constructs shows unique aggregation of cytoFTMT. (**a**) Heavy chain ferritin (FTH) (red) has well established cytoplasmic and nuclear distribution. (**b**) L*H ferritin chimera (red) shows diffuse cytosolic distribution. (**c**) Shows LacZ control vector and (**d**) cells without viral transduction (NV). Nuclei are stained with Hoechst blue. Scale bar = 20 μm.

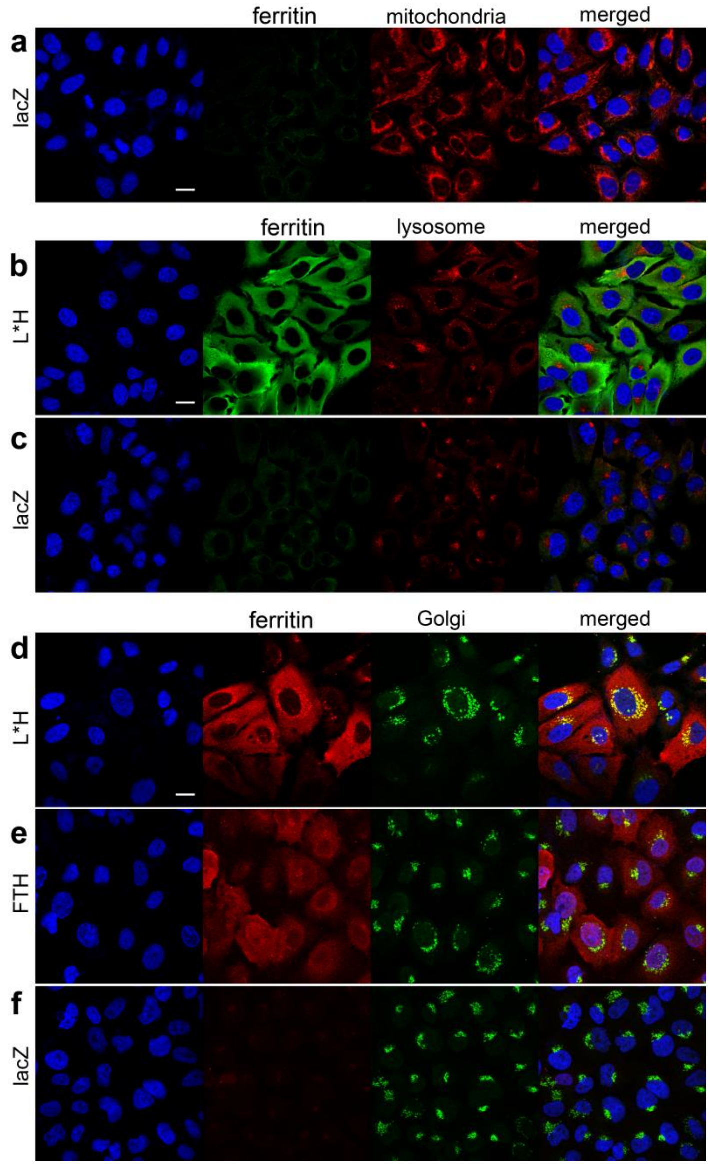

Figure S3. Confocal images of U2OS cells expressing control reporters and labeled cellular organelles. (**a**) Cells expressing lacZ control do not stain for ferritin and show normal mitochondrial staining (red). (**b**) L*H ferritin control (green) is not degraded by the lysosome (red). (**c**) Cells expressing lacZ control do not stain for ferritin, and show normal lysosomal stain (red). (**d**) In addition to abundant cytosolic distribution, some of the L*H ferritin (red) is processed in Golgi (green). (**e**) FTH (red) has diffuse cytosolic and nuclear staining and minimal colocalization with Golgi (green). (**f**) Cells expressing lacZ control do not stain for ferritin and show normal Golgi stain (red). Nuclei are stained with Hoechst blue. Scale bar = 20 μm.

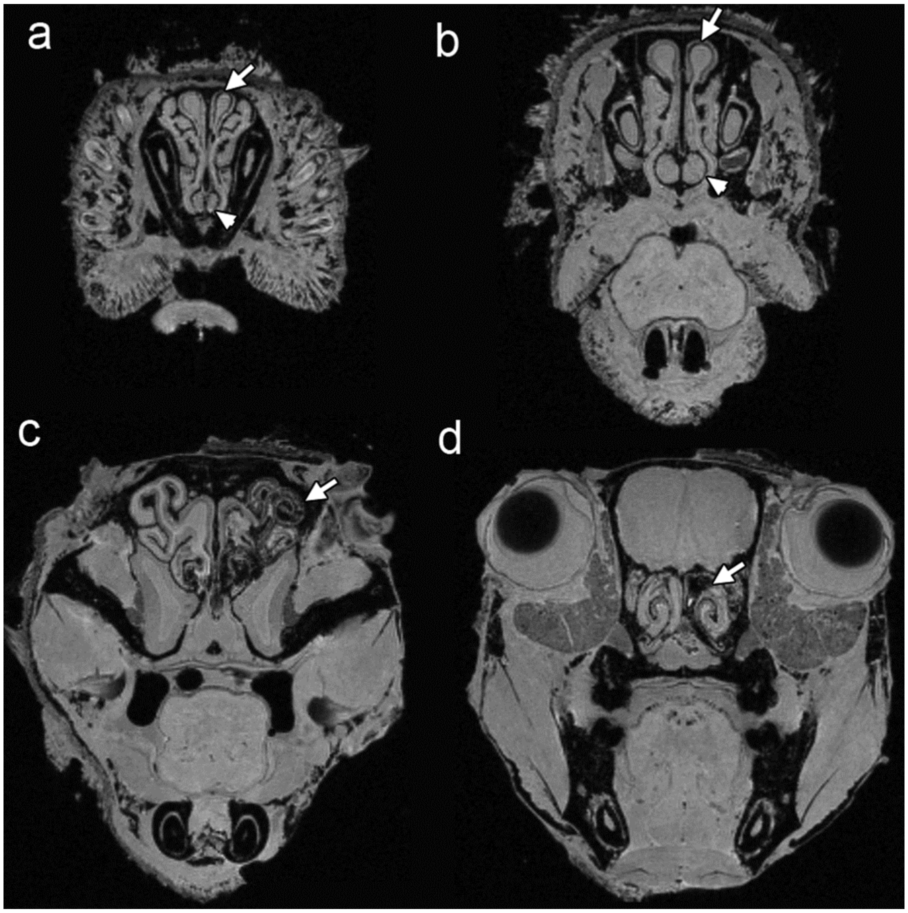

Figure S4. T_2* weighted MRI showing the extent of cytoFTMT reporter expression in mouse nasal passages. (**a**) Anterior portion of the epithelium showing minor contrast at the side of reporter (arrow) and no contrast at the vomeronasal organ (arrowhead). (**b**) Adjacent area of the epithelium, where arrow points to the small contrast change on the reporter side and arrowhead points to the vomeronasal organ. (**c**) A large portion of the olfactory epithelium (OE) with distinct cellular layers and a robust contrast change at the reporter gene expression site (arrow). (**d**) Posterior slice of the OE with arrow pointing at hypointensity due to local cytoFTMT expression.

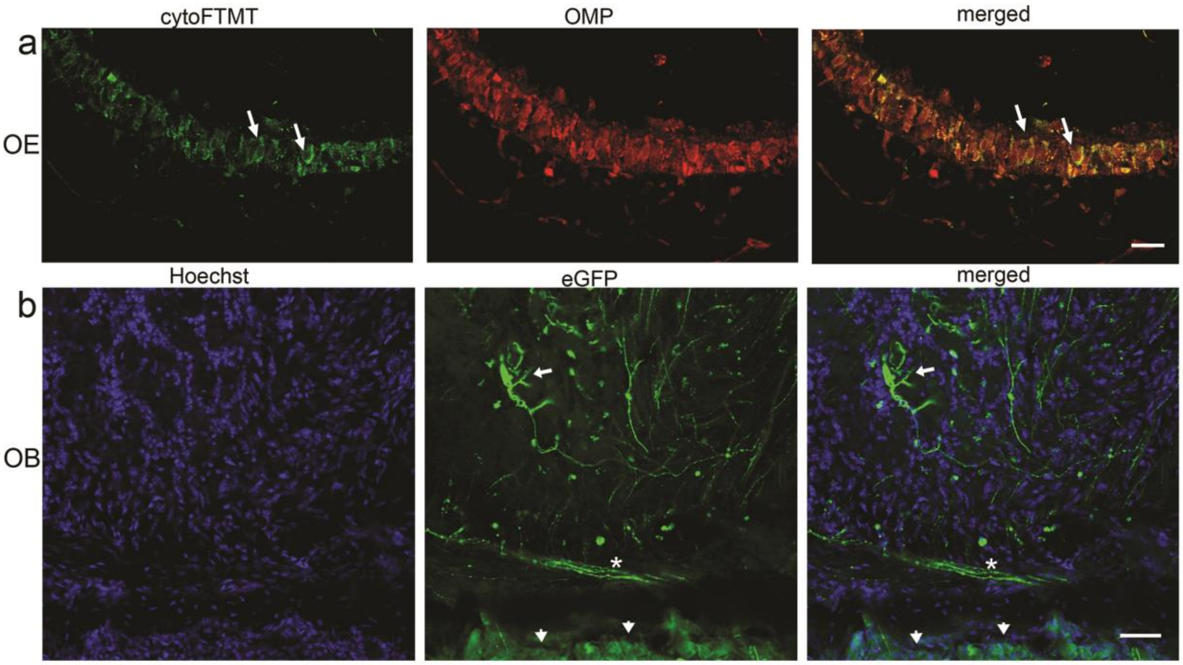

Figure S5. Immunohistochemistry of the olfactory epithelium (OE) and olfactory bulb (OB) of mice expressing the MRI reporter. (**a**) cytoFTMT (green) reporter forms aggregates (arrows) in cells positive for olfactory marker protein (OMP) (red). (**b**) The contralateral control side expresses eGFP in the OE (arrowheads). Green axonal fibers form the olfactory nerve (asterisk) and reach the OB to form synapses in the glomeruli (arrow).
